# Bupivacaine versus lidocaine analgesia for neonatal circumcision

**DOI:** 10.1186/1471-2431-5-12

**Published:** 2005-05-22

**Authors:** Orit C Stolik-Dollberg, Shaul Dollberg

**Affiliations:** 1 Department of Anesthesiology, Sheba Medical Center, Tel Hashomer, Israel; 2 Department of Neonatology, Tel Aviv Sourasky Medical Center, 6 Weizman Street, Tel Aviv 64239, Israel; 3 Sackler Faculty of Medicine, Tel Aviv University, Ramat Aviv, Tel Aviv, Israel

## Abstract

**Background:**

Analgesia for neonatal circumcision was recently advocated for every male infant, and its use is considered essential by the American Academy of Pediatrics. We compared the post-operative analgesic quality of bupivacaine to that of lidocaine for achieving dorsal penile nerve block (DPNB) when performing neonatal circumcision.

**Methods:**

Data were obtained from 38 neonates following neonatal circumcision. The infants had received DPNB analgesia with either lidocaine or bupivacaine. The outcome variable was the administration by the parents of acetaminophen during the ensuing 24 hours.

**Results:**

Seventeen infants received lidocaine and 19 received bupivacaine DPNB. Ten infants in the lidocaine group (59%) were given acetaminophen following circumcision compared to only 3 (16%) in the bupivacaine group (P < 0.01). Regression analysis showed that the only significant variable associated with the need for acetaminophen was the use of lidocaine (R^2 ^= 20.6; P = 0.006).

**Conclusion:**

DPNB with bupivacaine for neonatal circumcision apparently confers better analgesia than lidocaine as judged by the requirement of acetaminophen over the ensuing 24-hour period.

## Background

The issue of whether or not to circumcise a male infant one week after birth (when medically permissible) is essentially nonexistent in Israel. In an atmosphere of celebration and feasting, the procedure ("brit mila") has been carried out for over 4,000 years by non-physicians ("mohelim"). A mohel uses no anesthesia when performing the circumcision. The baby is given a few drops of sacramental wine and the mohel applies tight bandaging to the wound. The past few years, however, have witnessed a small but growing trend of young couples who seek to have their newborns circumcised by doctors using some kind of anesthesia in order to obviate the baby's pain and discomfort as well as their own anxiety. This cultural change has elicited interest among Israeli doctors in seeking optimal management of their newborn patients.

Anesthesia is not routinely administered for neonatal circumcision for a variety of reasons, among them the relatively short duration of the intervention, the perceived lack of importance of the pain, and concerns of toxicity from the medications [1]. It is now recognized that neonates are capable of both perceiving and exhibiting reproducible responses to pain, and that pain in neonates may have long-term effects (e.g., "pain memories") [2,3]. The routine use of analgesia during neonatal circumcision is now considered essential by the American Academy of Pediatrics [4].

A topical application of eutectic mixture of the local anesthetics, lidocaine and prilocaine (EMLA, Astra Pharma Inc. Sweden) had achieved considerable popularity for its ability to diminish pain associated with circumcision. In a review of the Cochrane database [5], however, its use was not shown to have any special advantage over other analgesic techniques with proven efficacy, such as regional nerve block with local anesthetic medications (e.g., lidocaine injection). We had been using lidocaine for dorsal penile nerve block (DPNB) in our service, and recently introduced bupivacaine because it has the important advantage of a longer duration of action compared to other local anesthetics [6]. In order to evaluate the clinical significance of this modification, we used the requirement of post-operative analgesia as a tool to assess long-term analgesic efficacy of analgesic medications in general [7], as well as specifically the requirement of acetaminophen after neonatal circumcision [8]. In the current study, we evaluated the duration of the long-term analgesic effect of bupivacaine in infants undergoing neonatal circumcision. We hypothesized that infants treated with bupivacaine will require less acetaminophen than infants treated with lidocaine.

## Methods

All male infants under the age of 2.5 months with normal penile anatomy were eligible for the study. By design, we excluded the infants with abnormal penile anatomy (e.g., hypospadias), those whose parents did not apply topical EMLA cream as instructed or when parents did not keep the scheduled follow-up appointment. The circumcision was performed by a board certified neonatologist and a board certified anesthesiologist in an office setting equipped with all necessary resuscitation equipment. Parents were instructed to apply approximately 2.5 grams of EMLA cream on the shaft of the penis and 1 cm around its base one hour prior to the appointed time for circumcision. In order to avoid absorption of the EMLA cream into the disposable diaper, parents were instructed to apply a 20 × 20 cm piece of household plastic wrap on the inside of the disposable diaper over the penis. Upon arrival to the office, the infants received 25–30 mg/kg acetaminophen, either orally or per-rectum, as preemptive analgesia (Acamoli syrup 120 mg/ml or Acamoli suppository 150 mg, Teva Medical, Petah Tikva, Israel). Oral doses of 40/kg body weight of acetaminophen have been used for post-operative pain treatment in infants [9]. Since acetaminophen has a large volume of distribution, a relatively large initial dose is required irrespective of whether treatment is administered orally or rectally. Tréluyer et al. found that the optimum oral dose was 30 mg/kg [10]. From 0.3–0.5 ml 30% sucrose-solution was given orally just prior to the analgesic injection [11]. All study infants received an injection of lidocaine 1% 6–8 mg as a subcutaneous ring block to the base of the penis by means of a hypodermic 27 G beveled needle (Becton Dickinson, Drogheda, Ireland) [12]. The infants were then injected with either lidocaine 1% (Ezracaine 1%, Rafa Laboratories, Jerusalem, Israel) 4–5 mg/kg (the lidocaine group which was comprised of infants who underwent the circumcision before May 18, 2003) or bupivacaine 1.5–2 mg/kg (Marcaine, Astra, Sweden), (the bupivacaine group consisting of infants who were circumcised after May 18, 2003) in a DPNB using a 25 G needle (Becton Dickinson) [13]. In order to avoid inadvertent intravenous injection, suction was applied to the syringe handle prior to injection, ensuring that the tip of the needle was not inside a blood vessel.

Circumcision was performed 4–5 minutes after analgesia administration. An additional dose of 0.3–0.5 ml 30% sucrose solution was dripped orally. The infants lay on a padded surface and circumcision was performed using the Mogen circumcision clamp (all procedures were carried out by S.D.) [14]. The infants were observed for adverse effects of analgesia or circumcision for at least 15 minutes and underwent a physical examination.

The parents were given an instruction sheet and verbally instructed by the physician to administer a dose of 30 mg/kg liquid acetaminophen 4 hours after the procedure if they subjectively felt that the infant displayed any signs they perceive as pain. One repeat dose was also recommended 4 hours after that if symptoms of pain or discomfort reappeared. The parents were instructed to contact the physician if pain persisted thereafter. A follow-up appointment was scheduled within 2–5 days after the circumcision, and the number of acetaminophen doses administered to the infants during the 24-hour period after the circumcision was routinely recorded. Consent was obtained from both parents.

Statistical methods included the t-test, Chi-square test, and regression analysis. Data are presented as mean ± SD. A P value of <0.05 was considered significant.

## Results

Thirty-eight consecutive infants who underwent neonatal circumcision between March 1 and June 30, 2003 were included in this study. Four other infants were excluded, 3 because the parents did not apply EMLA cream and one because his parents failed to keep the scheduled post-circumcision appointment. Of these 38 infants, 17 had DPNB with 1% lidocaine and 19 with 0.5% bupivacaine. There was no significant difference in birthweight (3065 ± 635 g vs. 3081 ± 570 g for the lidocaine and the bupivacaine groups, respectively), postnatal age (13.3 ± 7.2 days vs. 12.9 ± 8.3 days), or weight at circumcision (3256 ± 570 g vs. 3285 ± 517 g). None of the studied infants showed any clinical signs of cardiac or neurological toxicity after either analgesia.

Nine of the seventeen infants in the lidocaine group were given an additional dose of acetaminophen and another one received 2 doses (59%). In the bupivacaine group, 1 of the 19 infants received 1 dose and 2 were given two doses of acetaminophen (16%, χ^2 ^= 7.2; P < 0.01). None of the study infants was given more than 2 doses and none of the parents contacted the physician because they detected signs that their infant was experiencing pain or discomfort. Regression analysis, taking into account the need for acetaminophen as the dependent variable and the birthweight, current age, current weight and bupivacaine or lidocaine as the administered drug, showed that only the type of drug was significant in the regression model (R^2 ^= 20.6; P = 0.006).

## Discussion

Infants who were treated with bupivacaine injected as a DPNB had significantly better post-operative analgesia compared with lidocaine, as judged by their lower requirement of acetaminophen 24 hours after circumcision. Despite the recommendation to use 0.5% bupivacaine for DPNB in the previous edition of a major textbook of regional analgesia [15], a Medline search failed to identify any reports on the use of bupivacaine in DPNB for *neonatal *circumcision.

The use of bupivacaine for *pediatric *analgesia for circumcision was reported in two recently published studies performed on children older than 1 year. A study by Choi et al [6] compared the use of topical EMLA cream with bupivacaine in a randomized placebo-controlled manner and concluded that, despite no difference in the score obtained using a pain scale between the two groups, bupivacaine DPNB resulted in significantly longer analgesia. A second recently published study by Gauntlett [8] compared bupivacaine in DPNB with caudal bupivacaine with ketamine in 60 boys and reported no immediate adverse effects.

The use of bupivacaine may be potentially more hazardous than lidocaine in cases of accidental intravenous injection. Cardiac toxic effects of high doses or unintentional intravascular injection may lead to high plasma levels and related depression of the myocardium, decreased cardiac output, heart block, hypotension, bradycardia, ventricular arrhythmias and cardiac arrest [16]. Adverse central nervous system effects include restlessness, anxiety, dizziness, tinnitus, blurred vision or tremors and sometimes convulsions [16]. The safety of DPNB was recently studied in 3,909 pediatric patients undergoing circumcision. The authors did not report the type of medication and dosage that had been used and also failed to note if there had been any patients with accidental intravenous injection of the local anesthetic medication [17]. An additional study of neonatal circumcision with DPNB in 491 patients found that the only complication was bruising at the injection site in 11% of patients [18]. The type of drug used in this study was not specified in this study.

The mechanism of the reduced requirement of acetaminophen after bupivacaine injection is not clear. High protein binding, better lipid solubility and high pKa all contribute to the longer duration of analgesia, reported mean elimination half-life of 8.1 hours versus 3.2 hours of lidocaine [19]. It is possible that, in addition, the longer period of blocked nociception is sufficient to blunt the hypersensitization associated with persistent postoperative pain input. This secondary hyperalgesia is mediated in the spinal cord and contributes to postoperative pain [20].

Some potential limitations of our study should be mentioned. The data for this study were collected retrospectively and patient allocation was in a sequential fashion. This approach may introduce a potential bias: for example, while it is theoretically possible that the performance of the analgesia improved over time, thereby resulting in a better outcome in the bupivacaine group, the study was performed during a relatively short time frame and this drawback is not very likely. Secondly, the assessment of pain in the infants was done subjectively by the parents. Crying may have been misinterpreted by the parents as pain, but could have stemmed from other reasons, and could have been managed by some of the parents by modalities other than acetaminophen (e.g., feeding, diaper changing, rocking the infant). Since the parents in both groups were instructed in an identical manner (verbal and written instructions) to give acetaminophen if they felt that their son was in pain, this potential bias was probably not significant.

## Conclusion

Given the rarity of accidental intravenous injection and its longer analgesic effect, we conclude that bupivacaine is superior to lidocaine for DPNB when performing neonatal circumcision.

## Abbreviations

DPNB – Dorsal penile nerve block

## Competing interests

The author(s) declare that they have no competing interests.

## Authors' contributions

Both authors (Orit Stolik-Dollberg and Shaul Dollberg) 1) have made substantial contributions to conception and design, or acquisition of data, or analysis and interpretation of data; 2) have been involved in drafting the article or revising it critically for important intellectual content; and 3) have given final approval of the version to be published.

## Pre-publication history

The pre-publication history for this paper can be accessed here:


